# Direct Electrochemistry of Glucose Oxidase on Novel Free-Standing Nitrogen-Doped Carbon Nanospheres@Carbon Nanofibers Composite Film

**DOI:** 10.1038/srep09885

**Published:** 2015-05-06

**Authors:** Xueping Zhang, Dong Liu, Libo Li, Tianyan You

**Affiliations:** 1 State Key Laboratory of Electroanalytical Chemistry, Changchun Institute of Applied Chemistry, Chinese Academy of Sciences, Changchun 130022, P. R. China; 2 University of Chinese Academy of Sciences, Beijing 100049, P. R. China

## Abstract

We have proposed a novel free-standing nitrogen-doped carbon nanospheres@carbon nanofibers (NCNSs@CNFs) composite film with high processability for the investigation of the direct electron transfer (DET) of glucose oxidase (GOx) and the DET-based glucose biosensing. The composites were simply prepared by controlled thermal treatment of electrospun polypyrrole nanospheres doped polyacrylonitrile nanofibers (PPyNSs@PAN NFs). Without any pretreatment, the as-prepared material can directly serve as a platform for GOx immobilization. The cyclic voltammetry of immobilized GOx showed a pair of well-defined redox peaks in O_2_-free solution, indicating the DET of GOx. With the addition of glucose, the anodic peak current increased, while the cathodic peak current decreased, which demonstrated the DET-based bioelectrocatalysis. The detection of glucose based on the DET of GOx was achieved, which displayed high sensitivity, stability and selectivity, with a low detection limit of 2 μM and wide linear range of 12–1000 μM. These results demonstrate that the as-obtained NCNSs@CNFs can serve as an ideal platform for the construction of the third-generation glucose biosensor.

Direct electron transfer (DET) between biologically enzymes and electrode surface has attracted increasing interest because these investigations can contribute to the research of electron transfer mechanism in biological system and the development of biosensors and biofuel cells^1^,^2^,^3^. In particular, the DET-based analysis is crucial to the fabrication of the third-generation biosensors^4^. Unfortunately, it is extremely difficult to obtain the DET. Taking glucose oxidase (GOx) as an example, it rarely shows DET on bare electrode, because the redox center, flavine adenine dinucleotide (FAD), is embedded deeply in the protein shell^5^. In the past few years, the DET of GOx based on carbon nanotubes (CNTs) has been intensively studied and achieved significant progress^6^,^7^,^8^,^9^,^10^,^11^. However, recent studies have demonstrated that although CNTs are capable of promoting the DET of GOx, they cannot realize the DET-based glucose biosensing^12^,^13^. The noneffective electronic communication between enzyme and electrode surface has seriously limited the development of DET-based biosensors.

Carbon nanomaterials with excellent properties and unique structure have been widely utilized in the study of direct electrochemistry of enzymes^14^,^15^,^16^,^17^. For instance, Niwa’s group have prepared a novel carbon film via UV/ozone treatment, which realized the efficient DET of cytochrome c^18^. Recently, carbon nanospheres (CNSs), as promising and innovative carbon materials, are attracting wide attention. The surface of CNSs has many open edges and reactive “dangling bonds”, which are proposed to stimulate surface reactions^19^. However, the hydrophobicity of pristine carbon nanomaterials surface results in the poor biocompatibility, and relatively complex chemical functionalizations are always required^20^. Doping nitrogen into carbon nanomaterials is established to be an effective way to improve some special properties of host, such as the hydrophilicity, electron-donor ability and electrical conductivity^21^,^22^,^23^,^24^. Due to the favorable biocompatibility and electrocatalytic activity, nitrogen-doped CNSs (NCNSs) have been extensively applied to the sensing and electrocatalysis field^25^,^26^,^27^. Nevertheless, there are little reports about NCNSs being used in the study on DET of GOx. On the other hand, carbon nanofibers (CNFs) with large surface area and highly porous structure could be an ideal platform to support nanoparticles and immobilize biomolecules^28^,^29^,^30^. With this in consideration, we incorporate NCNSs into the body of CNFs for the expection of combining the large surface area of CNFs and the enhanced electrocatalytic activity of NCNSs to investigate the direct electrochemistry of GOx. Electrospinning can be an ideal method to prepare CNFs and CNFs-based composites by spinning polyacrylonitrile (PAN) or doped PAN with a subsequent thermal treatment^31^,^32^. This method is advantageous in the uniform dispersion of target objects, the high electrical conductivity of CNFs, and the highly porous and free-standing network structure of the resulting composites^33^.

In this work, we developed novel nitrogen-doped carbon nanospheres@carbon nanofibers (NCNSs@CNFs) composites by electrospinning polypyrrole nanospheres doped polyacrylonitrile (PPyNs@PAN) with subsequent thermal treatmernt for the investigation of DET and the DET-based glucose biosensing. Without any pretreatment, the as-obtained material can realize the DET of GOx and simultaneously possess the bioelectrocatalytic activity towards glucose. The detection of glucose based on the DET of GOx was achieved with high sensitivity, stability and selectivity. These results demonstrate that the as-obtained NCNSs@CNFs can serve as an ideal platform for the construction of the third-generation glucose biosensor. The proposed glucose biosensor can also work in O_2_-containing solutions with excellent analytical performances.

## Results

### Characterization of NCNSs@CNFs

**Figure 1:**
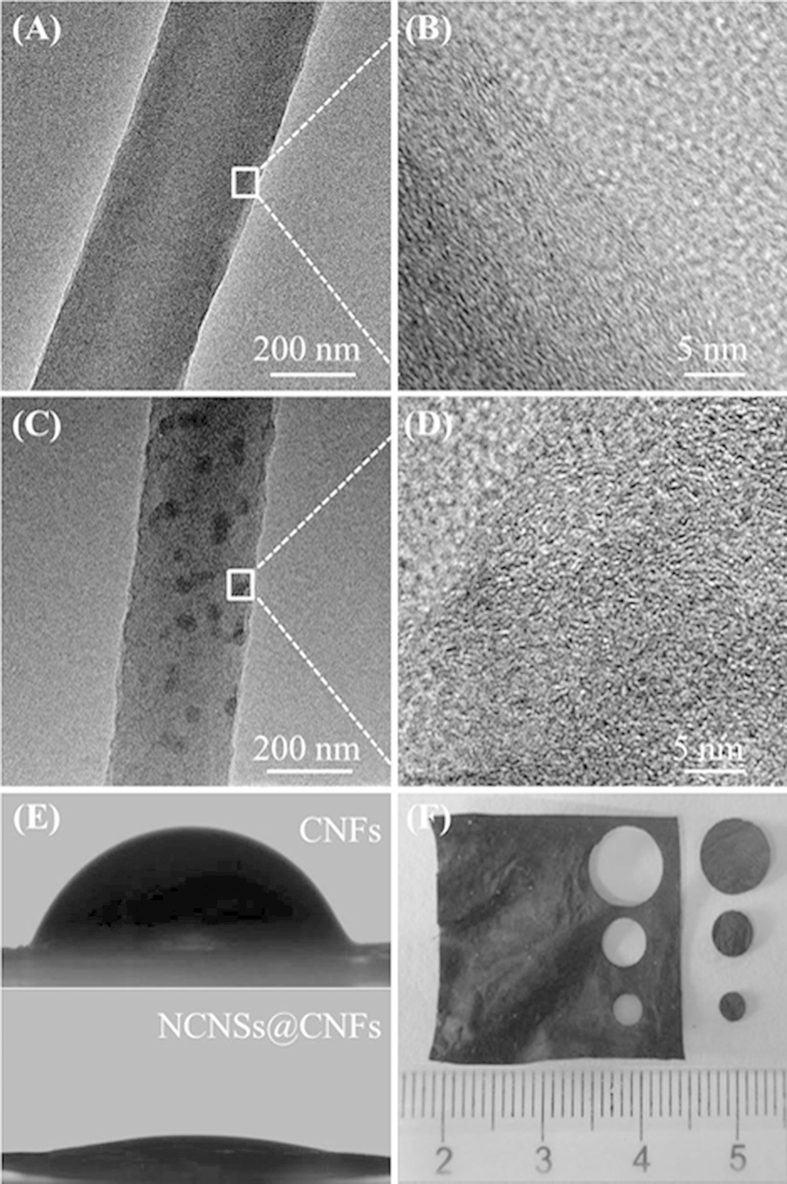
TEM images of CNFs (**A**) and NCNSs@CNFs (**C**). HRTEM images (**B**) and (**D**) are rectangular regions in (**A**) and (**C**). (**E**) Contact angles of CNFs and NCNSs@CNFs modified substrates. (**F**) Photograph of free-standing NCNSs@CNFs film.

The morphology of CNFs and NCNSs@CNFs were characterized by transmission electron microscopy (TEM). Compared with the bare CNFs with a diameter of 338 ± 12 nm (Fig. 1A), the as-prepared NCNSs@CNFs exhibit a relatively rough surface with a smaller diameter of 259 ± 28 nm (Fig. 1C). This could be attributed to the following two reasons: (1) the incorporated NCNSs are partially distributed on the surface of NCNFs, which can increase the surface roughness; (2) NH and NH_2_ radicals, which are derived from the decomposition of NH_3_ (mainly formed during the thermal treatment of PPyNSs^34^) can etch the surface, resulting in the decreased fiber diameter and increased surface roughnesss^35^. Furthermore, it can be obviously observed that NCNSs with an average diameter of 53 ± 9 nm (Fig. 1C and Supplementary Fig. S1A online) are relatively well-dispersed on the surface of CNFs or embedded within the matrix. The lattice fringes of CNFs can be clearly observed and the graphitic layers are generally aligned along the fiber axis according to the HRTEM image (Fig. 1B). However, the NCNSs@CNFs (Fig. 1D) exhibits a different structure with relatively vague lattice fringes, which is similar to the structure of NCNSs (Supplementary Fig. S1B online).

The hydrophilicity of the composite film was estimated by measuring the contact angle since the hydrophilicity of a carbon surface was essential to realize DET between an enzyme and the carbon electrode^36^. The contact angles of CNFs and NCNSs@CNFs were measured to be 68° and 14°, respectively (Fig. 1E), which demonstrated the better hydrophilicity of NCNSs@CNFs nanocomposite film. The improvement of the hydrophilicity could be ascribed to the multiple defects from nitrogen doping^37^. The improved hydrophilicity of NCNSs@CNFs may provide enhanced loading capacity of biomolecules as well as preserve their bioactivity.

**Figure 2:**
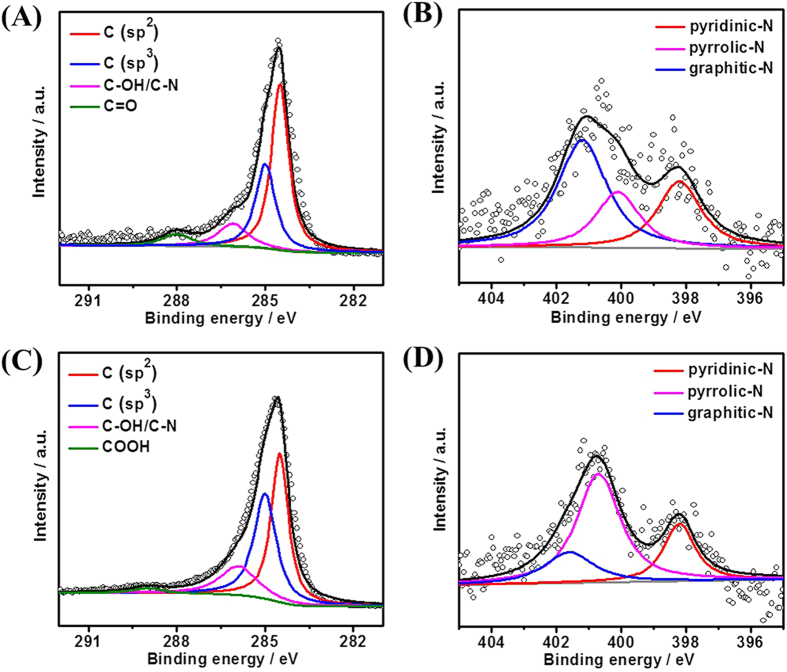
High-resolution XPS spectra of C1s and N 1s for CNFs (**A** and **B**) and NCNSs@CNFs (C and D).

X-ray photoelectron spectroscopy (XPS) was used to acquire the surface composition of the as-prepared materials. The C1s spectrum of NCNSs@CNFs (Fig. 2C) can be fitted into the same four component peaks as that of NCNSs (Supplementary Fig. S2A online) with binding energy of 284.5, 285.0, 286.1 and 289.0 eV, which are attributed to C (sp^2^), C (sp^3^), C-OH/C-N and -COOH, respectively^38^. In contrast, the C1s spectrum of CNFs (Fig. 2A) is different from that of NCNSs@CNFs in that the fourth peak is fitted into C = O with lower binding energy of 288.0 eV. The calculated percentage of different types of carbon are shown in Supplementary Table S1 online. Notably, compared with CNFs, the sp^3^/sp^2^ ratio of NCNSs@CNFs increases from 0.25 to 0.91, which is in consistent with the result of NCNSs (sp^3^/sp^2^ ratio=0.97). This fact implies that NCNSs have been successfully incorporated into the CNFs matrix. The decrease in the C (sp^2^) or increase in C (sp^3^) indicates that NCNSs@CNFs possess more structure defects than CNFs.

The N1s spectra for CNFs (Fig. 2B) and NCNSs@CNFs (Fig. 2D) both exhibit three main peaks, namely pyridinic-N, pyrrolic-N and graphitic-N with binding energy at 398.7 ± 0.3 eV, 400.4 ± 0.3 eV and 401.4 ± 0.3 eV, respectively^39^. The calculated percentage of the three types of N are shown in Supplementary Table S2 online. Compared with CNFs, the content of graphitic-N for NCNSs@CNFs decreased in a large extent (from 49.6 at. % to 18.7 at. %), while the content of pyrrolic-N increased significantly (from 22.9 at. % to 58.9 at. %). This phenomenon further confirms the successful doping of NCNSs into the CNFs matrix, because NCNSs possess a large content of pyrrolic-N and no graphitic-N exists, as evidenced by Supplementary Fig. S2B and Table S2 online. The formation of pyrrolic-N may be attributed to the reaction between the carbon host and H atoms or other H containing active species produced during the carbonization^40^. Previous studies have demonstrated that carbon materials with relatively high content of pyrrolic-N at the edges of graphene layers will display better charge mobility and donor-acceptor properties in comparison with pyridinic-N and graphitic-N^41^. Thus, NCNSs@CNFs may show better electrocatalytic activity than CNFs.

### Immobilization of GOx

The as-obtained NCNSs@CNFs display a unique self-supporting film structure with high processability, which can be directly tailored into designed shapes for electrode modification and GOx immobilization (Fig. 1F). Scanning electron microscopy (SEM) was applied to the analysis of NCNSs@CNFs composite film before and after the immobilization of GOx. When GOx was immobilized on the surface of NCNSs@CNFs, the GOx/NCNSs@CNFs exhibits similar three-dimensional incompact porous structure to NCNSs@CNFs (Supplementary Fig. S3A and B online). This uniform nanostructure may provide larger effective area for the immobilization of enzyme. Additionally, the excellent open structure of GOx/NCNSs@CNFs film could also enable the NCNSs@CNFs accessible to GOx molecules^37^.

**Figure 3:**
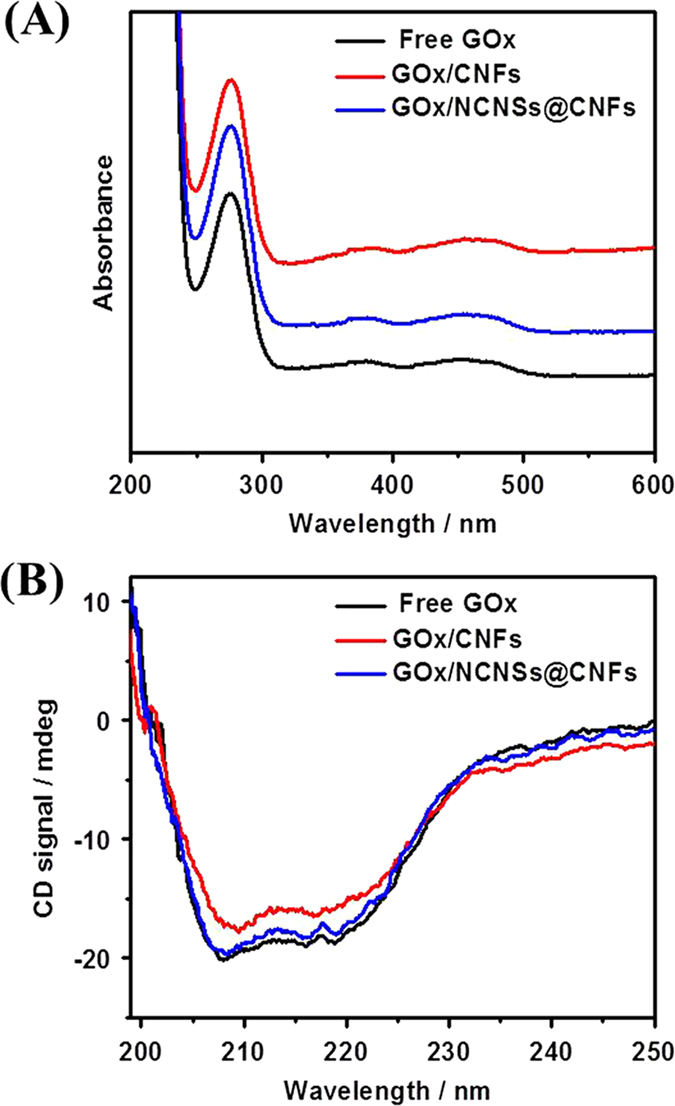
UV-vis (**A**) and far-UV CD (**B**) spectra of free GOx (black line), GOx/CNFs (red line) and GOx/NCNSs@CNFs (blue line) in 0.1 M PBS (pH 7.0) at room temperature.

Recent studies reported the denaturation of GOx upon its absorption on the nanostructured surface and the loss of enzyme function was usually related to the change of GOx structure^42^. UV-vis and Far-UV circular dichroism (CD) spectroscopy were used to examine the possible conformational changes of GOx during the modification process. As shown in Fig. 3A, for free GOx (black line), the UV-vis absorption peak at 277 nm is ascribed to the characteristic of polypeptide chains, and weak peaks at 380 and 454 nm represent the oxidized form of flavin groups in protein structure^43^. The position and shape of the absorption band for GOx on NCNSs@CNFs film (blue line, 277 nm, 380 nm and 454 nm) are the same as those of the free GOx, indicating that the GOx immobilized on NCNSs@CNFs film retains its native structure. However, when GOx was immobilized on CNFs, slight differences in the position and shape of the absorption band for GOx (red line, 277 nm, 382 nm and 455 nm) can be observed compared with those of free GOx.

The Far-UV CD spectra (Fig. 3B) further demonstrated the possible changes in the secondary structure of GOx during the immobilization procedure. The CD spectrum of free GOx is characterized by two negative bands at around 208 and 219 nm (black line). The peak intensity and position of GOx/ NCNSs@CNFs (blue line) are similar to those of the free GOx, while GOx/CNFs are a little different (red line). Hence, the NCNSs@CNFs film can serve as an ideal matrix for the immobilization of GOx by maintaining its original structure.

The electron transfer kinetics of [Fe(CN)_6_]^3−/4−^ redox couple at different modified electrodes were studied with EIS. The NCNSs@CNFs/GCE exhibited a much lower electron-transfer resistance (Supplementary Fig. S4B online, curve c, 17.5 Ω) in comparison with bare GCE (Supplementary Fig. S4A online, curve a, 200 Ω) and CNFs/GCE (Supplementary Fig. S4B online, curve a, 33.2 Ω), indicating that NCNSs@CNFs had high electrical conductivity. The improved conductivity of NCNSs@CNFs could be attributed to the introduction of NCNSs, whose electron-transfer resistance was extremely low (Supplementary Fig. S4B online, curve b). When GOx was fixed on the bare GCE, the electron-transfer resistance increased dramatically to 1620 Ω (Supplementary Fig. S4A online, curve b). However, the GOx/NCNSs@CNFs/GCE showed a much lower electron-transfer resistance (Supplementary Fig. S4A online, curve c, 382 Ω), indicating that the presence of NCNSs@CNFs can significantly accelerate the electron transfer.

### Direct Electrochemistry and Bioelectrocatalysis of GOx in O_2_-Free Solution

**Figure 4:**
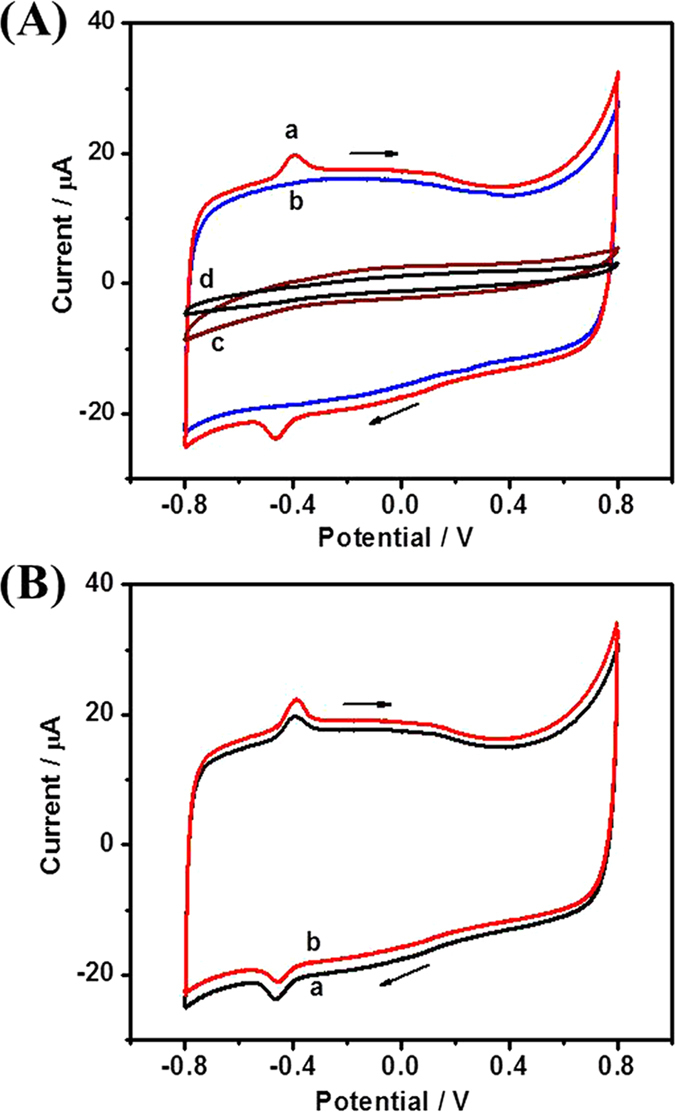
(**A**) CVs of GOx/NCNSs@CNFs (a), NCNSs@CNFs (b), GOx (c) modified GCEs and bare GCE (d); (**B**) CVs of GOx/NCNSs@CNFs/GCE in N_2_-saturated 0.1 M PBS (pH 7.0) without (a) and with (b) 1 mM glucose. Scan rate: 50 mV s^−1^.

GOx can be characterized by its electroactive enzyme cofactor FAD, which shows a two-electron coupled with two-proton redox reaction as FAD + 2H^+^ + 2e^− ^↔ FADH_2_^44^. GOx can oxidize glucose into D-glucono-1,5-lactone with the two protons and two electrons transferring from glucose to the GOx cofactor FAD to form FADH_2_. Supposing that FADH_2_ can be oxidized back to FAD, causing the restoration of GOx at the electrode surface with no need of any mediator, the process is termed as DET-based bioelectrocatalysis of GOx^4^. The above processes can be displayed as following^13^:
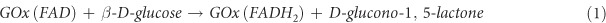

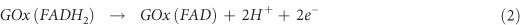
The DET between GOx and NCNSs@CNFs modified GCE was probed by CVs. In the O_2_-free solution, the as-prepared GOx/NCNSs@CNFs/GCE shows a pair of well-defined redox peaks at a formal potential of -0.43V (Fig. 4A, curve a), consistent with the typical electrochemical characteristics of GOx^37^. The control experiments of NCNSs@CNFs/GCE, GOx/GCE and GOx/CNFs/GCE (Fig. 4A, curve b, c and Supplementary Fig. S5 online) show no obvious response, indicating that the presence of both GOx and NCNSs@CNFs on the electrode surface is necessary to obtain the typical current response.

The DET-based bioelectrocatalysis of GOx towards glucose was investigated through CVs of GOx/NCNSs@CNFs/GCE in O_2_-free solution without and with 1mM glucose (Fig. 4B). Upon the addition of glucose, the anodic peak current increased (from 2.956 μA to 4.634 μA), while the cathodic peak current decreased (from 3.296 μA to 2.176 μA), which confirmed the DET-based bioelectrocatalysis^13^. As displayed in reaction 1 and 2, glucose is oxidized by GOx accompanied with the reduction of GOx (FAD) to GOx (FADH_2_). Simultaneously, GOx (FADH_2_) can be oxidized back to GOx (FAD) directly without any mediator. These reactions cause the changes of the GOx (FAD) redox peak currents and the restoration of GOx. For comparation, we also investigated the CVs of the FAD/NCNSs@CNFs/GCE in O_2_-free solution without and with 1 mM glucose (Supplementary Fig. S6 online). The addition of glucose has no effect on the peak currents of the FAD/NCNSs@CNFs/GCE, indicating that the FAD is the electroactive but not the enzymatically active part of GOx^12^.

**Figure 5:**
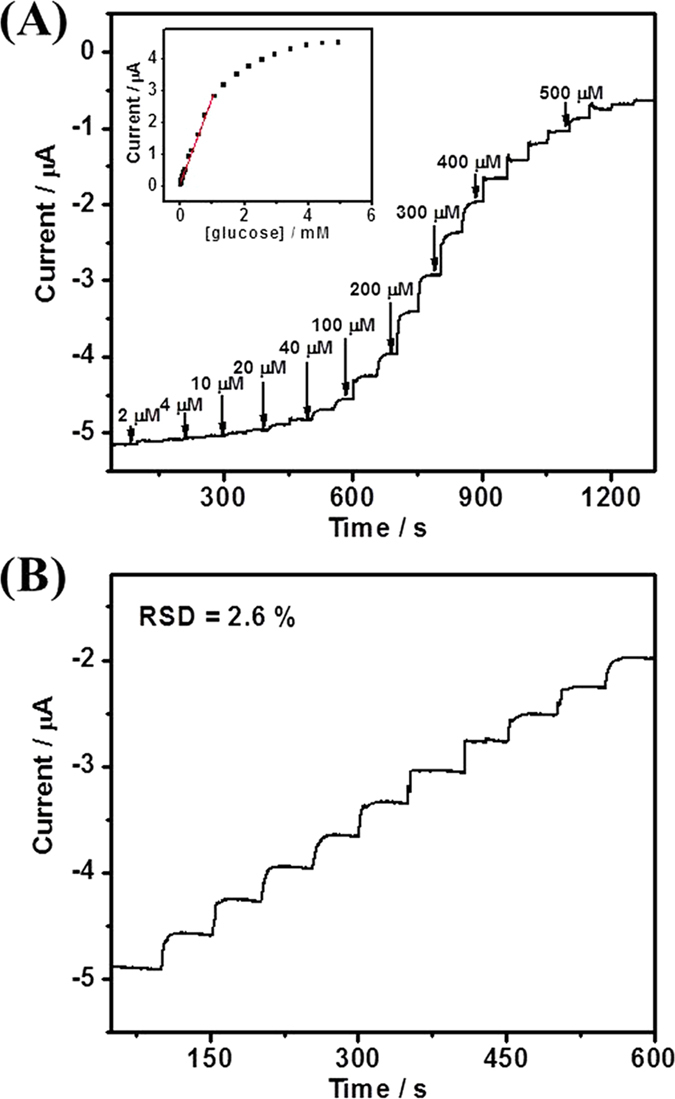
(**A**) Rotating disk chronoamperogram of GOx/NCNSs@CNFs in response to successive injections of glucose into N_2_-saturated 0.1 M PBS (pH 7.0) at the applied potential of -0.40 V (N_2_ constantly bubbled, rotating rate 1000 rpm). Inset: Calibration curve for glucose obtained at GOx/NCNSs@CNFs. (**B**) The current-time response curve for the successive addition of 0.1 mM glucose.

The DET-based glucose biosensing was investigated by rotating disk chronoamperometry at the applied potential of -0.40 V under O_2_-free condition (Fig. 5A). When N_2_-saturated glucose with different concentration was successively injected into the solution, obvious and fast current responses (within 5 s) were achieved. Under O_2_-free condition, the current response was ascribed to the DET-based bioelectrocatalytic oxidation of glucose by GOx. The as-proposed glucose biosensor displayed a linear range of 12-1000 μM (R^2^ > 0.996), with a sensitivity of 13.5 μA mM^−1^ cm^−2^ (based on the geometric area of the GC disk electrode) and a detection limit of 2 μM (S/N = 3) (Supplementary Fig. S7A online). The GOx/NCNSs@CNFs/GCE showed high stability towards the detection of glucose with a relative standard deviation (RSD) of 2.6% for ten continuous assays (Fig. 5B). The influence of interfering species on this biosensor was investigated with 0.4 mM AA, DA, and UA, which showed almost no interference towards glucose detection, indicating a high selectivity of the proposed method (Supplementary Fig. S8 online, black one).

### Bioelectrocatalysis of GOx in O_2_-Containing Solution

In O_2_-containing solution, the bioelectrocatalytic process of GOx towards glucose is different from that in O_2_-free solution, where glucose is oxidized by GOx into D-glucono-1, 5-lactone and oxygen is reduced by GOx to form H_2_O_2_:^12^
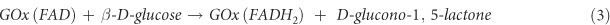

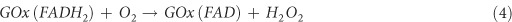


**Figure 6:**
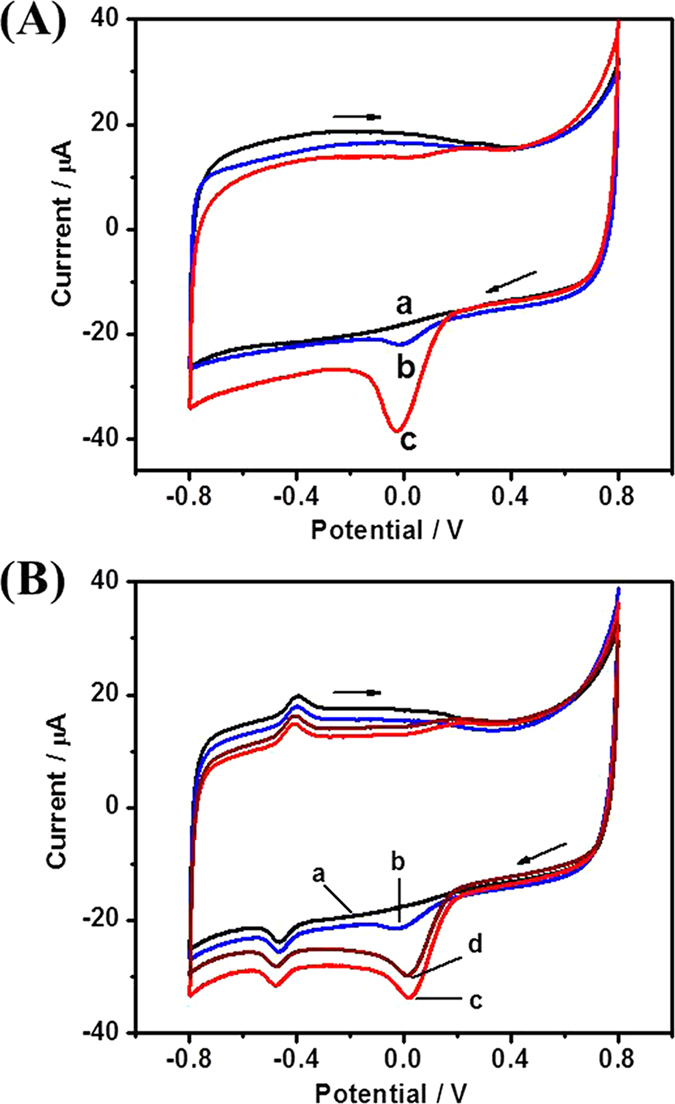
(**A**) CVs of NCNSs@CNFs/GCE in N_2_-saturated (a), air-saturated (b) and O_2_-saturated (c) 0.1 M PBS (pH 7.0); (**B**) CVs of GOx/NCNSs@CNFs/GCE in N_2_-saturated (a), air-saturated (b) 0.1 M PBS (pH 7.0) and O_2_-saturated 0.1 M PBS (pH 7.0) without (c) and with (d) 1 mM glucose. Scan rate: 50mV s^−1^.

The reduction of O_2_ at NCNSs@CNFs may affect the DET reaction of GOx and the glucose detection at negative potentials. Therefore, electrocatalysis of O_2_ reduction at NCNSs@CNFs/GCE was firstly explored. Compared with the CV in N_2_-saturated PBS (Fig. 6A, curve a), a reduction peak of O_2_ can be observed at -0.015 V in air-saturated PBS (Fig. 6A, curve b) and the peak current became larger in O_2_-saturated PBS (Fig. 6A, curve c). The excellent electrocatalysis of NCNSs@CNFs towards the reduction of O_2_ could be ascribed to: (1) the introduction of NCNSs, which could enhance surface reactions^19^, and (2) the relatively high content of pyrrolic-N and N-induced charge delocalization^35^.

Figure 6B compared the CVs of GOx/NCNSs@CNFs/ GCE in N_2_-saturated (curve a), air-saturated (curve b) and O_2_-saturated (curve c) PBS. As expected, GOx/NCNSs@CNFs/GCE exhibited strong electrocatalytic activity towards the reduction of O_2_, verified by a significant cathodic peak current at about 0 V, because O_2_ would compete with NCNSs@CNFs for the oxidation of FADH_2_. The incompact porous structure of NCNSs@CNFs film could facilitate the dissolved O_2_ molecules accessible to the immobilized GOx, enhancing the oxidation reaction of FADH_2_ by O_2_^4^. It was worth noting that the current due to the reduction of O_2_ did not overlap with the current peaks at -0.43V, which can simplify the analysis. When aliquot of 1 mM glucose was added into an oxygen-saturated 0.1 M pH 7.0 PBS, a significant decrease of the O_2_ reduction peak current was observed (curve d), which may be ascribed to the consumption of O_2_ from the natural enzymatic glucose oxidation. Such results implied that O_2_ can compete with the modified electrode for the oxidation of FADH_2_ and the immobilized GOx can still maintain their natural enzymatic activity.

**Figure 7:**
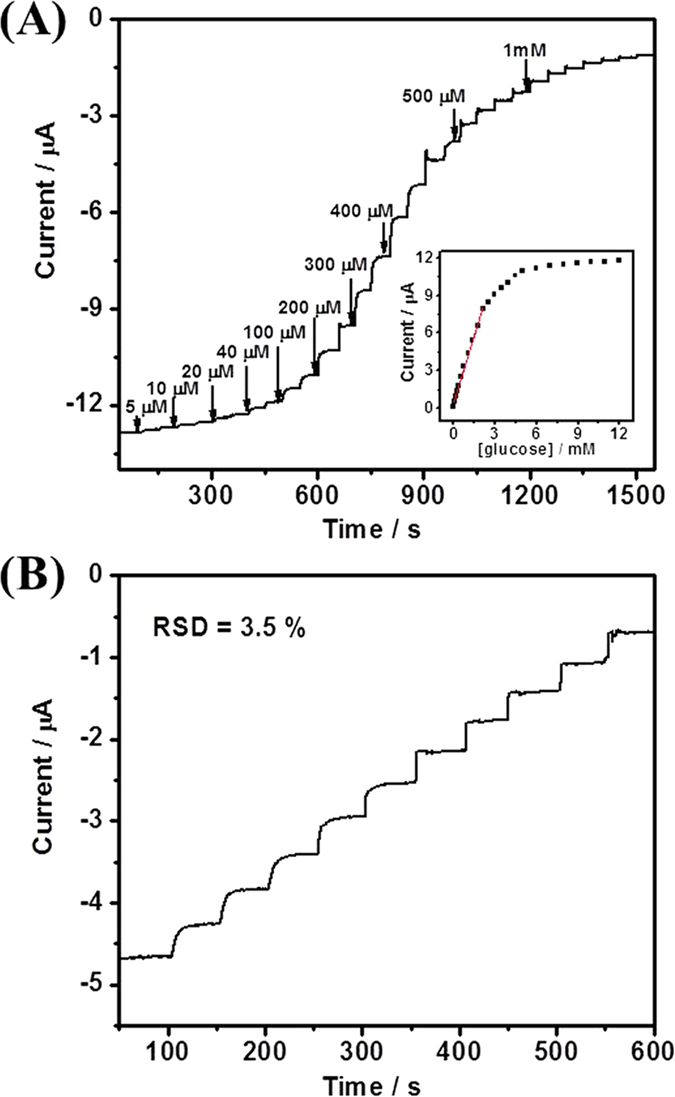
(**A**) Rotating disk chronoamperogram of GOx/NCNSs@CNFs in response to successive injections of glucose into air-saturated 0.1 M PBS (pH 7.0) at the applied potential of -0.40 V (air constantly bubbled, rotating rate 1000 rpm). (**B**) The current-time response curve for the successive addition of 0.1 mM glucose.

The detection of glucose was performed by rotating disk chronoamperometry at the applied potential of -0.40 V in air-saturated 0.1 M pH 7.0 PBS (Fig. 7A). Under O_2_-containing condition, the glucose biosensor showed a linear range of 10-2150 μM (R^2^ > 0.996), with a sensitivity of 18.4 μA mM^−1^ cm^−2^ and a detection limit of 4 μM (S/N = 3) (Supplementary Fig. S7B online). The linear range of glucose biosensing in air-saturated PBS was wider than that in O_2_-free PBS, suggesting that O_2_ was also involved in the biosensing process. The GOx/NCNSs@CNFs/GCE showed high stability towards the detection of 0.1 mM glucose with RSD of 3.5% for ten continuous assays (Fig. 7B). We also investigated the possible effect of interfering species on this biosensor with 0.4 mM AA, DA, and UA and obtained a high selectivity (Supplementary Fig. S8 online, red line).

## Discussion

The way to prepare NCNSs@CNFs composite film is rather simple and efficient, which can simultaneously combine the excellent electrocatalytic activity and high electrical conductivity of NCNSs with the large surface and porous open structure of CNFs. The as-obtained nanocomposites have favorable biocompatibility to maintain the natural activity of immobilized GOx. The highly porous open structure of NCNSs@CNFs can facilitate the DET between the active centers of GOx and the modified electrode, and enhance the mass diffusion of the matrixes, thus realizing the DET-based glucose biosensing. Compared with previously reported glucose biosensors (Table 1), GOx/NCNSs@CNFs/GCE shows relatively wide linear detection range, low detection limit and high sensitivity. All the above results demonstrate that the as-obtained NCNSs@CNFs composite film can serve as an excellent platform for the construction of the third-generation biosensors. Furthermore, we believe that the preparation method presented here is versatile to combine CNFs with other functional materials and thus to form a variety of CNFs-based multifunctional nanocomposites with free-standing structure.

In conclusion, we have prepared a novel NCNSs@CNFs composite film with free-standing structure and high processability by controlled thermal treatment of electrospun PPyNSs@PAN NFs. Without any pretreatment, the as-prepared material can serve as an ideal substrate for the immobilization of GOx and realize the efficient DET of GOx. More importantly, the DET-based bioelectrocatalysis towards glucose was achieved in O_2_-free solution. The detection of glucose based on the DET of GOx showed high sensitivity, stability and selectivity. The biosensor can also work on O_2_-containing solution, demonstrating that the immobilized GOx can still maintain their natural enzymatic activity.

## Methods

### Synthesis of NCNSs@CNFs Film

Firstly, polypyrrole nanospheres (PPyNSs) were synthesized according to the previous report^34^. The precursor PPyNSs@PAN composite nanofibers were obtained via electrospinning a DMF solution containing 10 wt. % PAN and 12 wt. % PPyNSs. And then, the thermal treatment of the electrospun composite nanofibers was conducted in a high temperature furnace and the carbonization process was performed in a device composed of two porcelain boats as reported previously^35^. Briefly, after stabilizing at 250 °C in air for 120 min, the samples were carbonized at 900 °C for 30 min in N_2_. The obtained material was marked as NCNSs@CNFs. For comparison, CNFs was prepared via the same process without PPyNSs.

### Electrode Preparation and Enzyme Immobilization

Prior to the modification, the working electrodes were polished carefully with 0.3 and 0.05 μm alumina powder, respectively, followed by rinsing thoroughly with ethanol and double-distilled water. The obtained NCNSs@CNFs film was cut into a designed shape with a diameter of 3.0 or 5.0 mm and directly fixed on the surface of GCE (NCNSs@CNFs/GCE) or GC disc electrode. Then, 6.0 or 16 μL of 2.0 mg mL^−1^ GOx solution was dropped onto the surface of NCNSs@CNFs/GCE or NCNSs@CNFs modified GC disc electrode, respectively. The modified electrodes were allowed to dry at 4 °C in a refrigerator to obtain the GOx/NCNSs@CNFs modified electrodes. To maintain the stability of modified electrodes, a drop of 3.0 or 8.0 μL 1% Nafion solution was cast on the membrane before electrochemical measurements. All enzyme-modified electrodes were stored in 0.1 M PBS (pH 7.0) at 4 °C in a refrigerator when not in use.

### Characterizations and measurements

Scanning electron microscopy (SEM) was carried out on a PHILIPS XL-30 field-emission scanning electron microscope at an accelerating voltage of 15 kV. Transmission electron microscope (TEM) images were obtained from a TECNAI F20 field-emission transmission electron microscope operated at 200 kV. The static water contact angles were measured at room temperature with a DSA10-MK2 contact angle goniometer (Krüss, Germany) employing drops of pure deionized water. X-ray photoelectron spectra (XPS) were performed on an ESCALAB-MKII X-ray photoelectron spectrometer with an excitation source of Al Kα radiation. The UV-vis spectra were measured on UV mini 1240 (Shimadzu, Japan). The circular dichroism (CD) spectra in the far-UV (with the range from 190 to 260 nm) were obtained on a JASCO J-820 spectropolarimeter using a 1 cm quartz cuvette.

Electrochemical impedance spectroscopy (EIS) was operated on AUTOLAB in 5 mM [Fe(CN)_6_]^3−/4−^ with 0.1 M KCl as supporting electrolyte. The cyclic voltammograms (CVs) and rotating disk chronoamperometry were performed on a CHI 832C electrochemical workstation (Chenhua Instrument, China) using a 3.0-mm diameter glassy carbon electrode (GCE) or 5.0-mm GC disk electrode (Pine Instruments) as the working electrode, respectively. A platinum plate and Ag/AgCl (saturated KCl) served as the counter and reference electrode, respectively. All the experiments were carried out in phosphate buffer solution (PBS) (0.1 M, pH 7.0) at room temperature. The nitrogen-saturated or oxygen-saturated buffer was prepared by purging them with high purity nitrogen or oxygen for 30 min and maintained a nitrogen or oxygen blanket above the solution during a measurement.

## Author Contributions

X. Z. designed the study, performed the main experiments and wrote the main manuscript. T. Y., D. L. and L. L. provided general coordination of the study and revised the manuscript. All authors have reviewed the manuscript.

## Additional Information

**How to cite this article**: Zhang, X. *et al.* Direct Electrochemistry of Glucose Oxidase on Novel Free-Standing Nitrogen-Doped Carbon Nanospheres@Carbon Nanofibers Composite Film. *Sci. Rep.* **5**, 09885; doi: 10.1038/srep09885 (2015).

## Supplementary Material

Supporting Information Supplementary Figure S1-S8

## Figures and Tables

**Table 1:** Comparison of the analytical performances of different glucose biosensors.

**Glucose biosensor**	**Linear Range (μM)**	**Detection Limit (μM)**	**Sensitivity (μA mM**^**−1**^ **cm**^**−2**^)	**Ref.**
GOx/NCNSs@CNFs/GCE^a^	12−1000	2	13.5	This work
GOx/NCNSs@CNFs/GCE^b^	10−2150	4	18.4	This work
GOx-X^c^-APTES^d^/BDD^e^	15−400	10	--	4
GNSs^f^-Nafion-GOx/GCE	200−1400	--	3.4	2
GOx/N-CNTs^g^/GCE	Up to 6500	24	11.0	13
GOx/CNx-MWNTs^h^/GCE	Up to 1020	10	13.0	37
PEI/{GOx/PEI}_3_/CNT/GCE	Up to 300	--	106.57	9
GOx/cage-like-PbS/Nafion/GCE	50−1450	10	11.2	43

^a^Under O_2_-free condition.

^b^Under O_2_-containing condition.

^c^Glutaraldehyde.

^d^3-Aminopropyltriethoxysilane.

^e^Boron-doped diamond.

^f^Graphite nanosheets.

^g,h^Nitrogen-doped carbon nanotube.
