# Supplementary material for: Direct Electrochemistry of Glucose Oxidase on Novel Free-Standing Nitrogen-Doped Carbon Nanospheres@Carbon Nanofibers Composite Film
Source: Sci Rep. 2015 May 6;5:9885. doi: 10.1038/srep09885 (PMC4421824; doi:10.1038/srep09885)
Supplement: Supporting Information — Supplementary Figure S1-S8 [file srep09885-s1.pdf]

Supplementary information

Direct Electrochemistry of Glucose Oxidase on Novel Free-Standing Nitrogen-Doped Carbon Nanospheres@Carbon Nanofibers Composite Film

Xueping Zhang,[1,2] *Dong Liu*,[1] *Libo Li*[1,2] and *Tianyan You**,[1]

[1] State Key Laboratory of Electroanalytical Chemistry, Changchun Institute of Applied Chemistry, Chinese Academy of Sciences, Changchun 130022, China.

[2] University of Chinese Academy of Sciences, Beijing 100049, China.

* youty@ciac.jl.cn (T. Y. You), Tel: +86-431- 85262850, Fax: +86-431-85262850.

1. TEM and HRTEM images of NCNSs

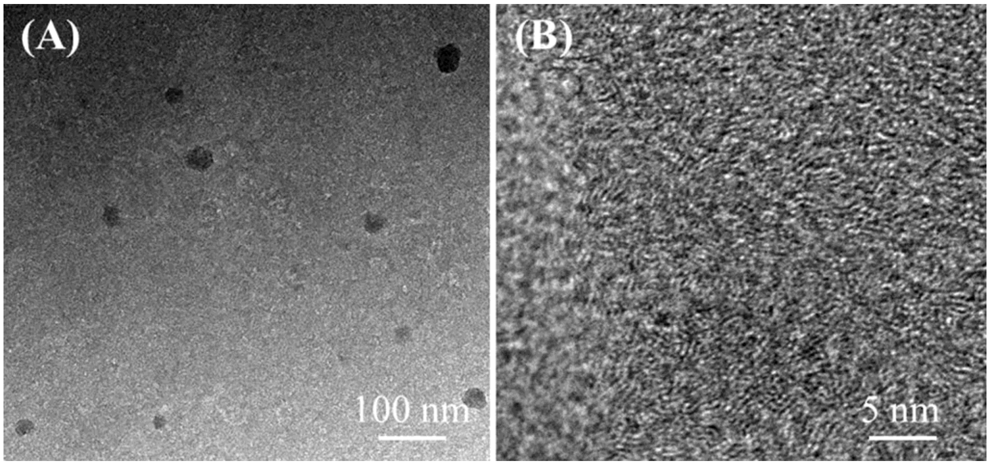

Supplementary Figure S1. TEM (A) and HRTEM (B) images of NCNSs.

2. XPS spectra of NCNSs

The deconvolution of high-resolution XPS spectra over the C1s and N1s region for NCNSs is shown in Fig. S2A and B, respectively. The calculated percentages of different types of carbon and nitrogen functional groups are shown in Table S1 and Table S2, respectively.

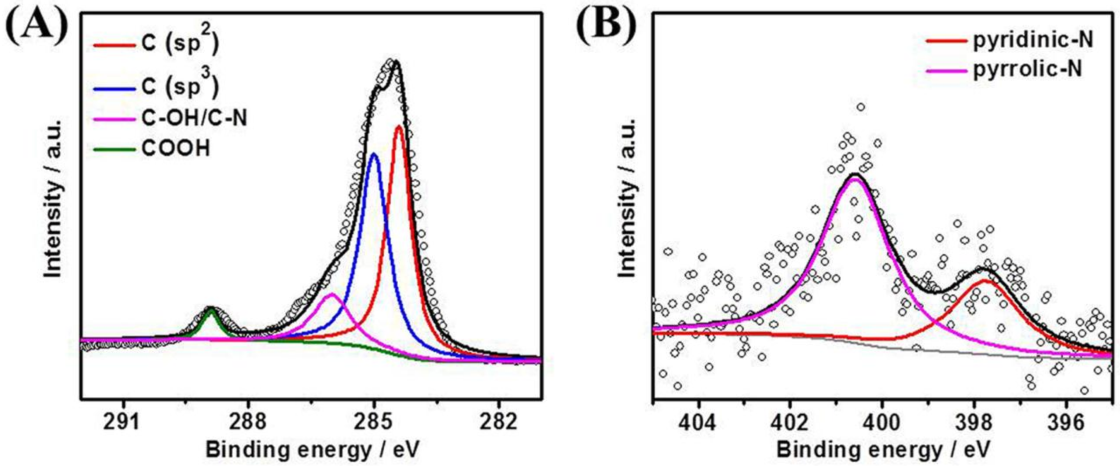

Supplementary Figure S2. High-resolution XPS spectra of C1s (A) and N 1s (B) for NCNSs.

Supplementary Table S1. Results of the fits of the XPS C1s spectra, values given in at. % of total intensity.

Sample	C (sp^2)	C(sp^3,defects)	C-OH/C-N	C=O	-COOH
CNFs	49.2	12.3	8.7	29.8	--
NCNSs@CNFs	41.9	38.1	17.9	--	2.4
CNSs	41.9	40.8	13.5	--	3.8

Suplementary Table S2. Results of the fits of the XPS N1s spectra, values given in at. % of total intensity.

Sample	pyridinic-N	pyrrolic-N	graphitic-N
CNFs	27.5	22.9	49.6
NCNSs@CNFs	22.4	58.9	18.7
NCNSs	31.3	68.7	0

3. Characterization of NCNSs@CNFs and GOx/NCNSs@CNFs

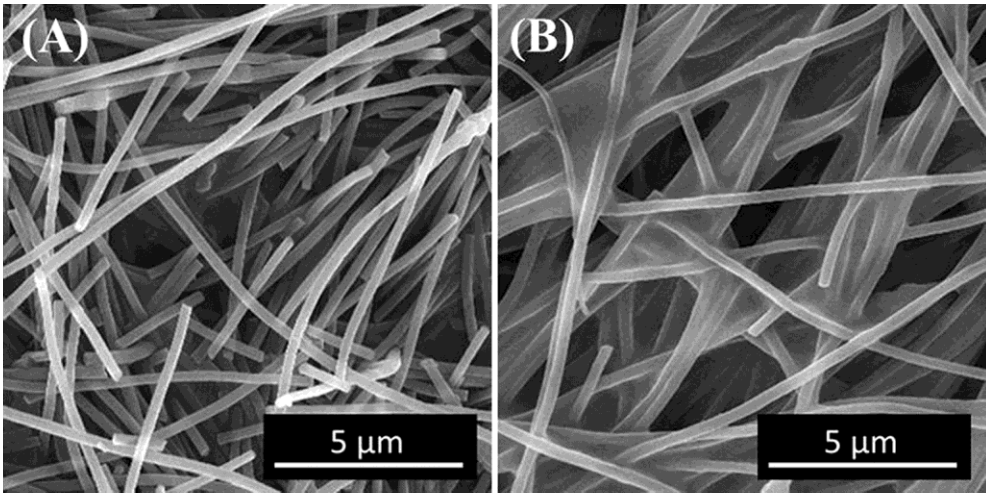

Supplementary Figure S3. SEM images of NCNSs@CNFs (A) and GOx/NCNSs@CNFs (B).

4. EIS analysis of the modified electrodes

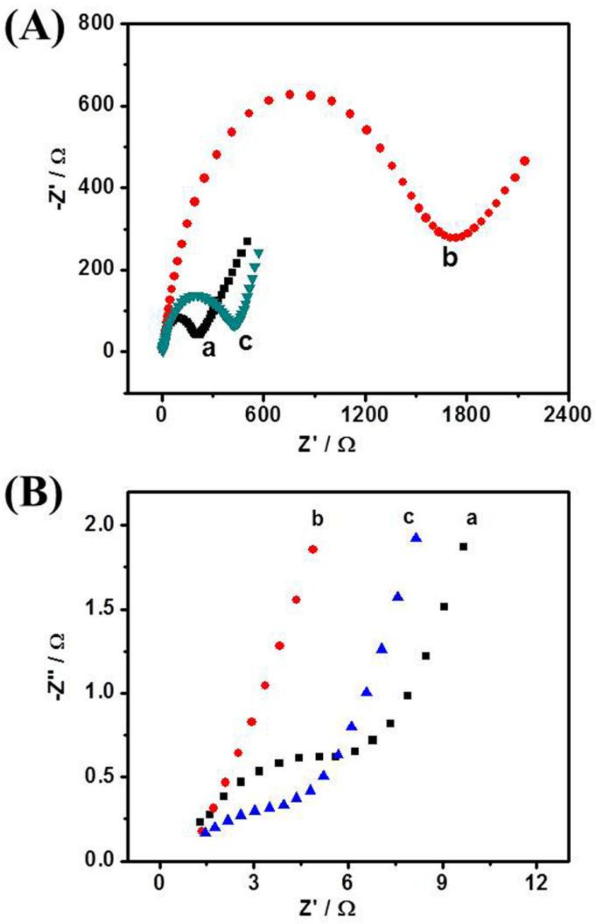

Supplementary Figure S4. (A) Electrochemical impedance spectra of bare GCE (a), GOx (b) and GOx/NCNSs@CNFs (c) modified GCEs; (B) Electrochemical impedance spectra of CNF (a), NCNSs (b) and NCNSs@CNFs (c) modified GCEs in 5 mM $[Fe(CN)_6]^{3-/4-}$ with 0.1 M KCl as supporting electrolyte.

.

5. Analytical Performance

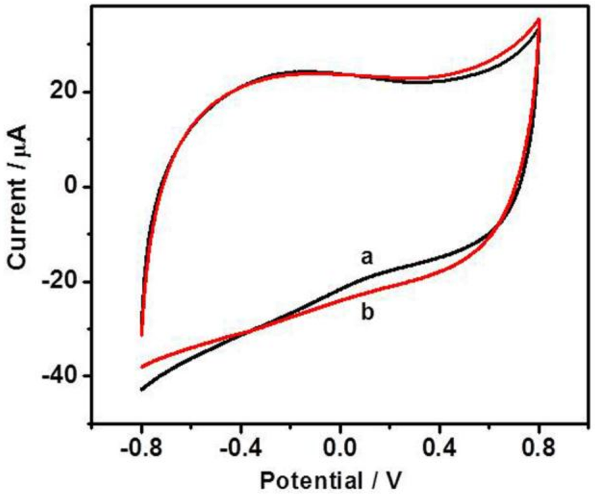

Supplementary Figure S5. CVs of CNFs (a) and GOx/CNFs (b) modified GCEs in nitrogen-saturated 0.1 M PBS (pH 7.0). Scan rate: 50 mV s^{-1}.

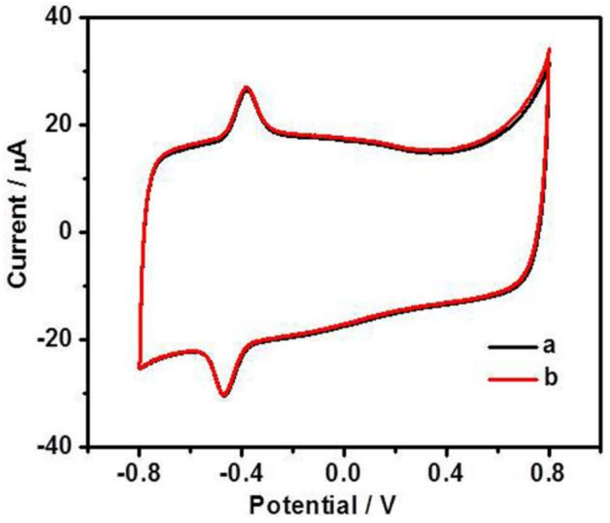

Supplementary Figure S6. CVs of FAD/NCNSs@CNFs/GCE in nitrogen-saturated 0.1 M PBS (pH 7.0) without (a) and with (b) 1 mM glucose. Scan rate: 50 mV s^{-1}.

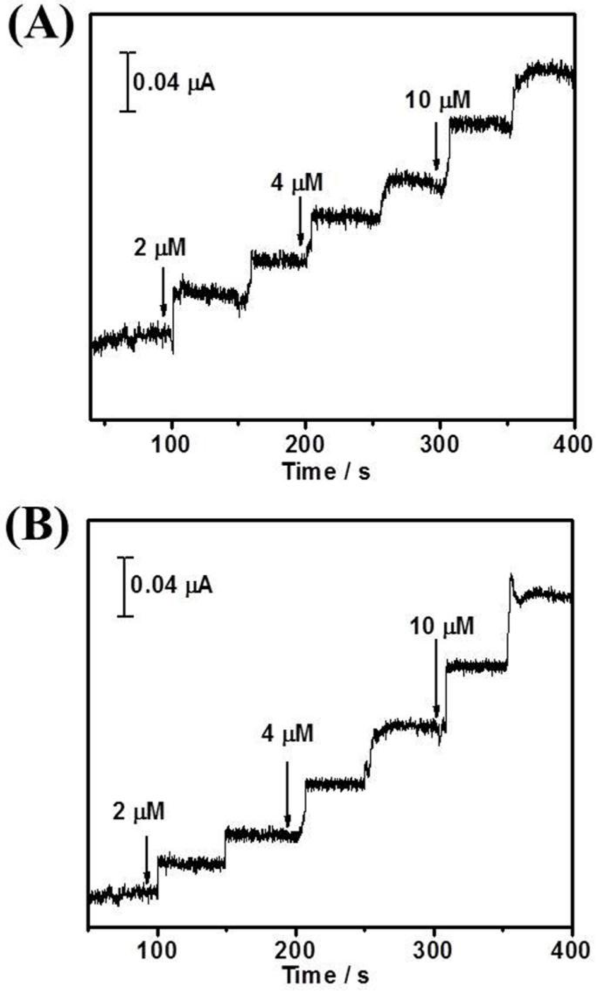

Supplementary Figure S7. Rotating disk chronoamperograms of GOx/NCNSs@CNFs in response to different concentrations of glucose in (A) nitrogen-saturated and (B) air-saturated 0.1 M PBS (pH 7.0) at the applied potential of -0.40 V (N_2 or air constantly bubbled, rotating rate 1000 rpm).

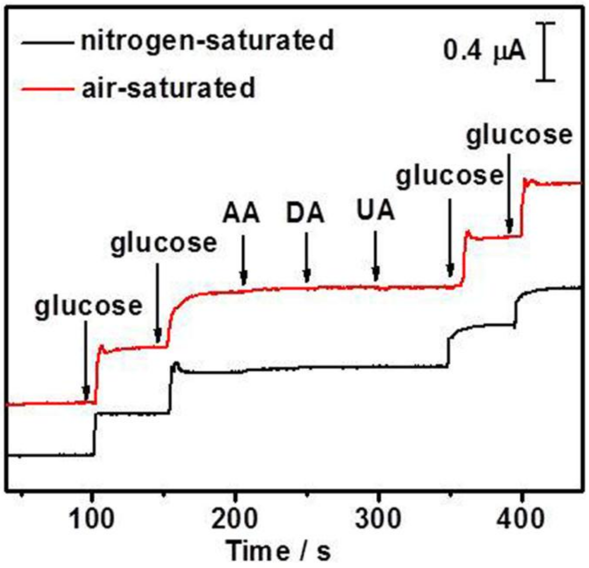

Supplementary Figure S8. Rotating disk chronoamperograms of GOx/NCNSs@CNFs in response to the addition of 0.1 mM glucose, 0.4 mM AA, 0.4 mM DA, 0.4 mM UA, and 0.1 mM glucose into nitrogen-saturated (black line) and air-saturated (red line) 0.1 M PBS (pH 7.0) at the applied potential of -0.40 V (N_2 or air constantly bubbled, rotating rate 1000 rpm).
